# Enhancing automated fracture detection in paediatric wrist X-rays with paired and unpaired cast suppression methods

**DOI:** 10.1007/s11548-026-03595-2

**Published:** 2026-03-19

**Authors:** Stanley A. Norris, Daniel Carrion, Franko Hržić, John R. Zech, Sergio Uribe, Mohamed K. Badawy

**Affiliations:** 1 Monash Radiology, Monash Health, Melbourne, Australia; 2 Department of Medical Imaging and Radiation Sciences, School of Primary and Allied Health Care, Faculty of Medicine, Nursing and Health Sciences, Monash University, Melbourne, Australia; 3 Centre for Artificial Intelligence and Cybersecurity, Faculty of Engineering, University of Rijeka, Rijeka, Croatia; 4 Faculty of Engineering, University of Rijeka, Rijeka, Croatia; 5 Department of Radiology, Columbia University Irving Medical Center, New York, USA

**Keywords:** Wrist X-rays, Cast suppression, Fracture detection, Generative adversarial networks (GANs), Artificial intelligence

## Abstract

**Purpose:**

Casts in follow-up wrist X-rays reduce diagnostic image quality, complicating the assessment of fracture healing. This study developed cast suppression models using real unpaired data and synthetic paired data and investigated their impact on automated fracture detection performance in paediatric wrist X-rays.

**Methods:**

A cast suppression model was developed using data generated with unpaired image-to-image translation methods. A published CycleGAN model was repurposed to generate a synthetic paired dataset from an institutional collection of 31,001 X-rays. This dataset was used to train Pix2Pix cast suppression models, using three architectures—U-Net 256, 512, and 1024. Models were evaluated using structural similarity index measure (SSIM), mean-squared error (MSE), and peak signal-to-noise ratio (PSNR). A fracture detection model was trained on a publicly available dataset of 20,327 paediatric wrist X-rays. This model was used to evaluate CycleGAN and Pix2Pix-based cast suppression by testing performance across training sets with varying cast prevalence, defined as 100, 50, 25, and 0% of the naturally occurring cast proportion in the training dataset.

**Results:**

Within Pix2Pix models, the U-Net 1024 configuration performed best across all metrics (SSIM = 0.957, MSE = 22.2, PSNR = 34.7 dB) and was selected for subsequent fracture detection experiments. Cast suppression improved fracture detection only in models trained without any cast exposure, where Pix2Pix preprocessing increased mAP@0.5 by 4%, from 0.823 (0.008) to 0.859 (0.004), but reduced mAP@0.5 by 1–2% and mAP@0.5:0.95 by 3–4% in models that included cast images during training. Pix2Pix consistently outperformed CycleGAN-based cast suppression across both evaluation metrics (mAP@0.5 and mAP@0.5:0.95), with comparisons assessed using bootstrap resampling.

**Conclusion:**

The Pix2Pix model outperformed CycleGAN-based cast suppression for fracture detection preprocessing. However, cast suppression only improved performance in models trained without cast images and reduced performance otherwise. These findings indicate that cast suppression effectiveness depends critically on the downstream model’s training data composition.

**Supplementary Information:**

The online version contains supplementary material available at https://doi.org/10.1007/s11548-026-03595-2.

## Introduction

Hand and wrist trauma is a major driver of emergency department workload, accounting for approximately 5% of presentations and incurring significant economic costs through treatment, follow-up imaging, and productivity loss [1–3]. Radiography is routinely used to diagnose, evaluate, and follow up wrist fractures due to its accessibility, affordability, and low radiation exposure [4]. Damaged limbs often require stabilisation using splints or casts made of resin, fibreglass, or plaster [5]. However, the presence of immobilisation devices introduces artefacts that obscure anatomical detail, complicate diagnostic interpretation in follow-up assessments, and decrease the performance of fracture detection models [6].

Although the clinical need for image quality improvement in medical imaging has driven much interest in artificial intelligence-based solutions [7–10], relatively few studies have explored the suppression of immobilisation devices in extremity radiographs. To date, attempts to address this clinical problem have used CycleGAN models, a form of generative adversarial network (GAN) capable of generating or modifying images by translating them between different visual domains [11]. Hržić et al. showed that CycleGAN models could be used to suppress cast artefacts in paediatric wrist X-rays [12], Lee et al. showed that CycleGAN-based splint suppression improved the performance of an automated fracture detection model on adult wrist X-rays [13], and Norris et al. showed that CycleGAN-generated wrist X-rays could improve the diagnostic confidence of radiologists reporting on difficult cases [14]. Despite such promising results, existing studies face important methodological limitations.

The use of CycleGAN models introduces risks of anatomical distortion or hallucination that can be difficult to detect or quantify [15]. First, the absence of paired cast and cast-free wrist X-rays has forced previous studies to rely on indirect, histogram-based similarity metrics, such as histogram correlation [12], histogram intersection [16, 17], Chi-squared distance [18], and Hellinger distance [19]. These measures capture only global intensity similarity, ignoring spatial structure and potential alterations to important anatomical regions or fracture appearance. Consequently, they provide limited insight into diagnostic image quality or the clinical safety of suppressed images. Second, although radiologist assessment provides a clinically meaningful alternative, it is resource-intensive and difficult to scale. Systematically evaluating cast suppression in this way requires radiologists to manually review large numbers of images, which is impractical in typical clinical research settings where radiologists’ time is limited [20–22]. Consequently, prior studies have been constrained either by indirect metrics that lack clinical relevance or by radiologist assessments that are difficult to implement at scale.

An alternative approach for validating cast suppression has been to examine its effect on automated fracture detection performance, as demonstrated by Lee et al. [13]. While this strategy offers an objective measure linked to diagnostic tasks, it also introduces limitations. Fracture detection models are susceptible to the composition of their training datasets, which may underrepresent X-rays containing immobilisation devices. As a result, improvements observed after cast suppression may reflect the model’s lack of exposure to cast images during training rather than genuine enhancements in anatomical visibility or diagnostic utility. In such cases, the benefit of suppression may be confounded by training-data bias, complicating the interpretation of prior findings.

To address these gaps, we generated a synthetic paired dataset using an unpaired CycleGAN model and used it to train supervised Pix2Pix cast suppression models, enabling direct pixel-wise evaluation of reconstruction accuracy. We then evaluated both paired (Pix2Pix) and unpaired (CycleGAN) models as preprocessing steps for fracture detection, explicitly controlling the cast composition of the detection model’s training data. In this work, fracture detection is used as a technical surrogate task to objectively evaluate the impact of cast suppression.

## Methods

This study was classified as a retrospective quality assurance activity and was reviewed by the Monash Health Research Office (Research Support Services). The project was confirmed to be exempt from Human Research Ethics Committee review and approved via an alternate governance pathway, in accordance with the National Health and Medical Research Council (NHMRC) Ethical Considerations in Quality Assurance and Evaluation Activities (2014) guideline.

### Data curation and hardware

**Fig. 1:**
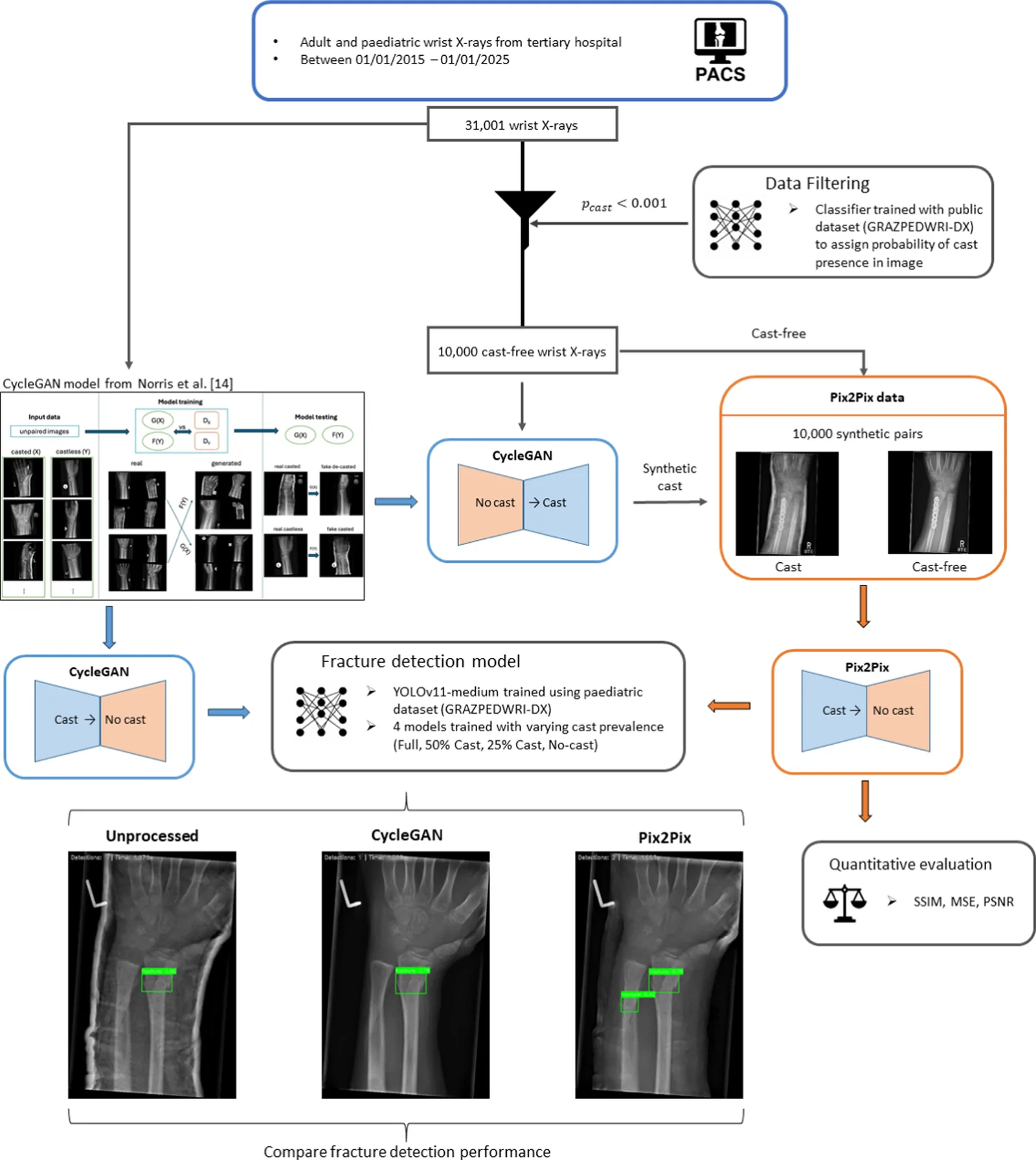
Overview of the study workflow, including image classification for data filtering, cast suppression modelling, and downstream fracture detection evaluation

All adult and paediatric wrist X-rays acquired at a tertiary hospital between 1 January 2015 and 20 January 2025 were identified through a picture archiving and communication system (PACS) query. This resulted in 31,001 X-rays, hereafter referred to as the MELBWRI-DX dataset. The institutional dataset is not publicly available due to privacy regulations. Images were exported in PNG format, converted to 8-bit grayscale, and resized to 1024 × 1024 pixels with zero padding. A deep learning–based classifier was used to assign each image a probability of cast presence. The model was trained on the publicly available GRAZPEDWRI-DX dataset [23] and achieved 99.5% accuracy on its test set. It was then applied to the MELBWRI-DX dataset to identify cast-free images. The 10,000 images with the lowest cast-probability scores were retained for subsequent modelling. Training details and performance metrics of the classification model are provided in the supplementary information. No images from MELBWRI-DX were used for fracture detection model training or testing, ensuring complete separation between cast suppression development and downstream detection experiments (Table 1). An overview of the study design is provided in Fig. 1.

**Table 1:** Dataset-model mapping used in this study

Dataset	Used for	Models involved	Study population
MELBWRI-DX (31,001 X-rays)	Cast synthesis and suppression	CycleGAN, Pix2Pix	Adult and paediatric
GRAZPEDWRI-DX (20,327 X-rays)	Fracture detection	YOLOv11-medium	Paediatric

The classifier and all subsequent models in this study were implemented in Python (v3.10) using PyTorch 2.4 and CUDA 12 and were trained on two NVIDIA Tesla P40 GPUs with 24 GB of VRAM each.

### Cast synthesis and Pix2Pix training

**Fig. 2:**
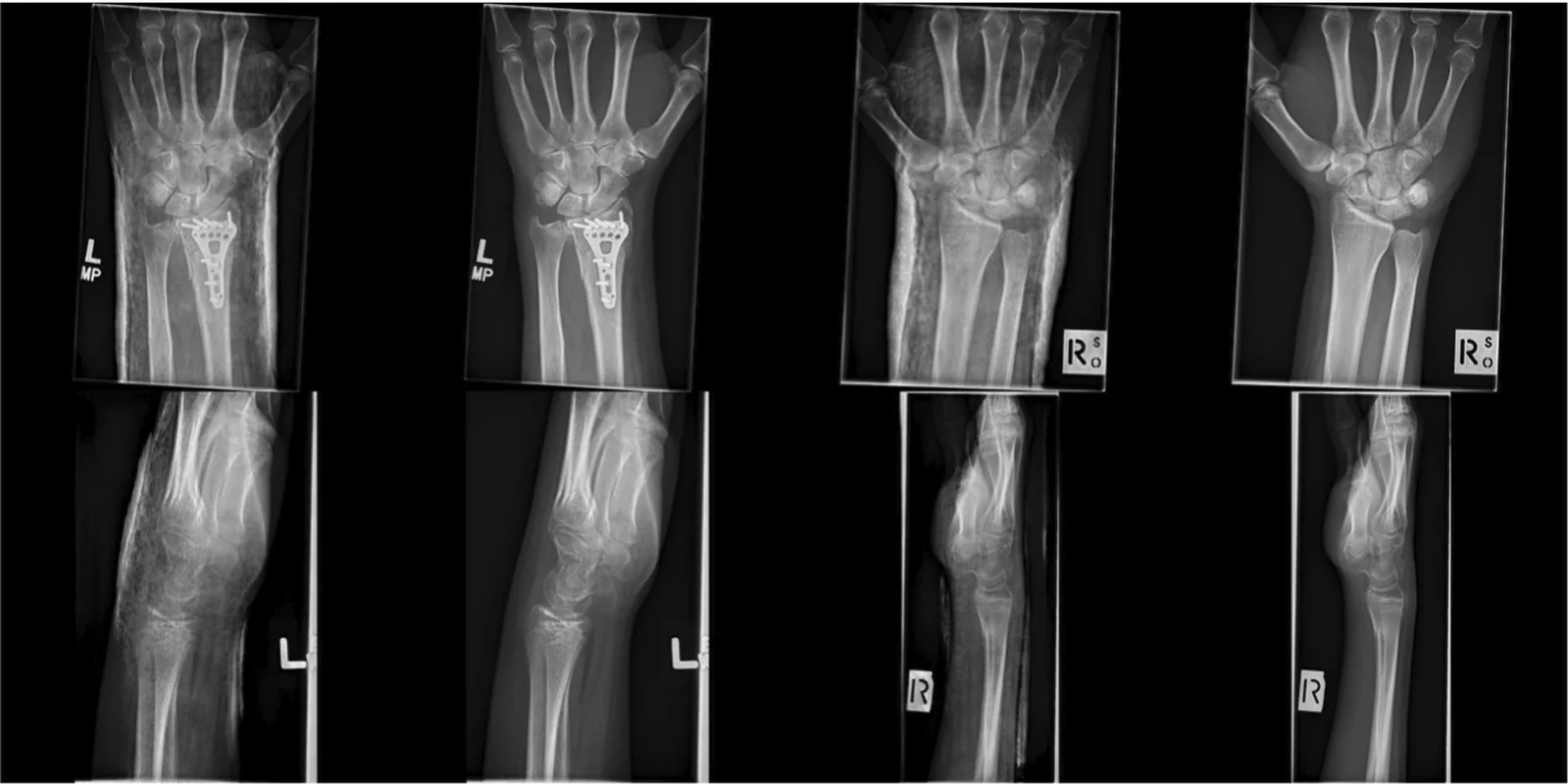
Representative sample of synthetic paired wrist X-rays, showing CycleGAN-generated cast images (left) and corresponding original cast-free images (right)

A previously published CycleGAN model [14], developed from the same institutional dataset, was used to synthesise cast artefacts on cast-free wrist X-rays. The pretrained generator was applied to the classifier-selected MELBWRI-DX images to create paired samples comprising an original, cast-free X-ray and a synthetic cast version (Fig. 2), yielding 10,000 aligned image pairs.

The paired dataset was randomly split into 8000 training pairs and 2000 test pairs (80:20). Following the standard Pix2Pix supervised image-to-image translation framework [24], we trained three models using U-Net generator architectures of increasing capacity (U-Net 256, U-Net 512, and U-Net 1024). These architectures were selected to compare performance across networks with differing receptive fields and feature extraction depth. All models were trained for 100 epochs with a constant learning rate of 0.002 for the first 50 epochs, followed by linear decay over the remaining 50 epochs. The Adam optimiser [25] was used with β_1_ = 0.5 and a batch size of 1.

### Quantitative Pix2Pix model evaluation

The three Pix2Pix configurations were evaluated using the held-out test set of 2000 paired X-rays. Structural similarity index measure (SSIM), peak signal-to-noise ratio (PSNR), and mean-squared error (MSE) were computed for each generated cast-suppressed X-ray against its corresponding cast-free reference. These metrics jointly assess perceptual similarity, signal fidelity, and pixel-wise reconstruction accuracy. Metrics were calculated across the full test set for each U-Net generator (256, 512, and 1024), and results are reported as median values with interquartile ranges (IQR). While these quantitative measures do not reflect clinical diagnostic quality, they provide an objective basis for comparing model architectures and selecting the most appropriate configuration for subsequent fracture detection evaluation [26–28].

### Fracture detection model training

The publicly available dataset GRAZPEDWRI-DX [23], comprising 20,327 annotated paediatric wrist trauma X-rays, was used to train fracture detection models, while MELBWRI-DX was used only for cast suppression model development. The GRAZPEDWRI-DX dataset was split into training, validation, and test sets using a stratified 70:15:15 split, respectively. Patient-level separation was maintained to avoid any potential for data leakage, and cast prevalence was balanced across training, validation, and test sets (~ 30% with cast, ~ 70% without casts).

To examine how cast suppression interacts with training data composition, we trained four YOLOv11-medium fracture detection (FD) models differing only in the number of cast images included (Table 2). The YOLOv11 architecture was selected as a well-established, state-of-the-art object detection architecture at the time of this study, offering a strong balance between detection performance and computational efficiency. The ‘FD-100’ configuration used the complete training set. The ‘FD-50’, ‘FD-25’, and ‘FD-0’ configurations progressively reduced the number of cast-containing X-rays, by 50, 75, and 100%, respectively, while keeping all 10,162 cast-free images. This design enabled systematic evaluation of how varying levels of cast exposure during training influence the effect of cast suppression at inference.

**Table 2:** Training dataset composition for the four YOLOv11-medium fracture detection model configurations, where all datasets derive from the baseline GRAZPEDWRI-DX training split

Model	Cast-free images	Cast images	Total images
FD-100	10,162	4,045	14,207
FD-50	10,162	2,022	12,184
FD-25	10,162	1,011	11,173
FD-0	10,162	0	10,162

All models were trained using identical hyperparameters. Training settings, data augmentation details, and learning curves are provided in the supplementary information. Demographic and composition details of the held-out test set used for evaluation are summarised in Table 3.

**Table 3:** Demographic and composition details of the test dataset used to evaluate fracture detection models

Characteristic	n	(%)
Total images	3,043	–
Unique patients	916	–
Gender Male Female	1,781 1,262	58.5 41.5
Age (years)	11 (0–19)	–
Laterality Left Right	1,669 1,374	54.8 45.2
Cast present No Yes	2,157 886	70.9 29.1
Fracture visible No Yes	1,059 1,984	34.8 65.2

Values are presented as counts with percentages unless otherwise indicated. Age is reported as median and range in years

### Fracture detection performance evaluation

Model performance was quantified using mean average precision (mAP), a standard object detection metric that evaluates detection accuracy across varying confidence thresholds [29]. We report mAP at an intersection-over-union (IoU) threshold of 0.5 (mAP@0.5) and the averaged metric across IoU thresholds from 0.5 to 0.95 in increments of 0.05 (mAP@0.5:0.95). An IoU threshold defines the minimum overlap between a predicted and ground-truth bounding box required for a correct detection. During evaluation, the confidence threshold was set to 0.001 to retain all candidate predictions, and non-maximum suppression (NMS) was applied using an IoU threshold of 0.6 to remove duplicate bounding boxes with substantial overlap.

To assess how cast suppression preprocessing influences fracture detection performance, we evaluated the four trained fracture detection models (‘FD-100’, ‘FD-50’, ‘FD-25’, and ‘FD-0’) under three preprocessing conditions. These conditions were: (1) original (no preprocessing), (2) CycleGAN cast suppression (using the previously published model [14]), and (3) Pix2Pix cast suppression (using the model with the best quantitative metrics). Preprocessing was applied only to the 886 cast-containing images in the test set, while the 2,157 cast-free images were left unchanged for all conditions. This design allowed direct comparison of how each suppression method affects fracture detection performance for the same training dataset compositions.

### Statistical analysis

For the quantitative evaluation of the Pix2Pix models, normality of SSIM, PSNR, and MSE distributions—and their paired differences—was assessed using the Shapiro–Wilk test. Because all models were evaluated using the same set of reference targets, the data were paired across the three U-Net configurations (256, 512, and 1024). For non-normally distributed paired data with three related groups, overall differences were assessed using the Friedman test [30]. Where significant effects were identified, pairwise comparisons were performed using Wilcoxon signed-rank tests with the Bonferroni correction [31].

For the cast suppression preprocessing experiment, the performance variability of each fracture detection model was estimated using a nonparametric bootstrap. Twenty bootstrap test sets were generated by sampling with replacement from the original 3,043-image test set, each preserving the same sample size (*N* = 3,043). The number of bootstrap resamples was selected based on an empirical convergence analysis performed to ensure stable estimation of performance variability; visual inspection of running medians and interquartile ranges for mAP@0.5 and mAP@0.5:0.95 demonstrated stable performance estimates beyond approximately 20 iterations (Supplementary Fig. 5S). All preprocessing conditions (‘Original’, ‘Pix2Pix’, ‘CycleGAN’) were applied to the same bootstrap samples, yielding paired performance estimates. Within each fracture detection model, the three preprocessing conditions were compared using Wilcoxon signed-rank tests, with Bonferroni adjustment applied to the three possible pairwise comparisons. All statistical analyses were performed in Python (v3.12.4).

## Results

### Quantitative Pix2Pix model evaluation

**Fig. 3:**
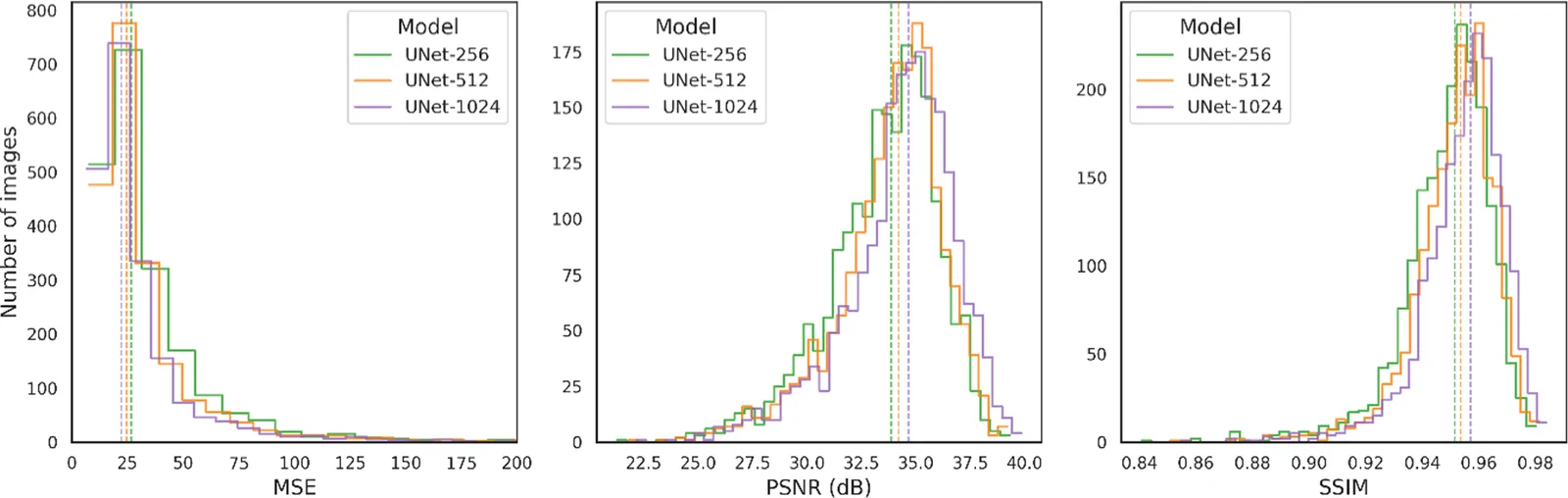
Distribution of MSE, PSNR, and SSIM values for the 2,000 held-out test image pairs, comparing Pix2Pix U-Net generator configurations (U-Net 256, U-Net 512, and U-Net 1024). Vertical dashed lines indicate median values for each configuration

**Fig. 4:**
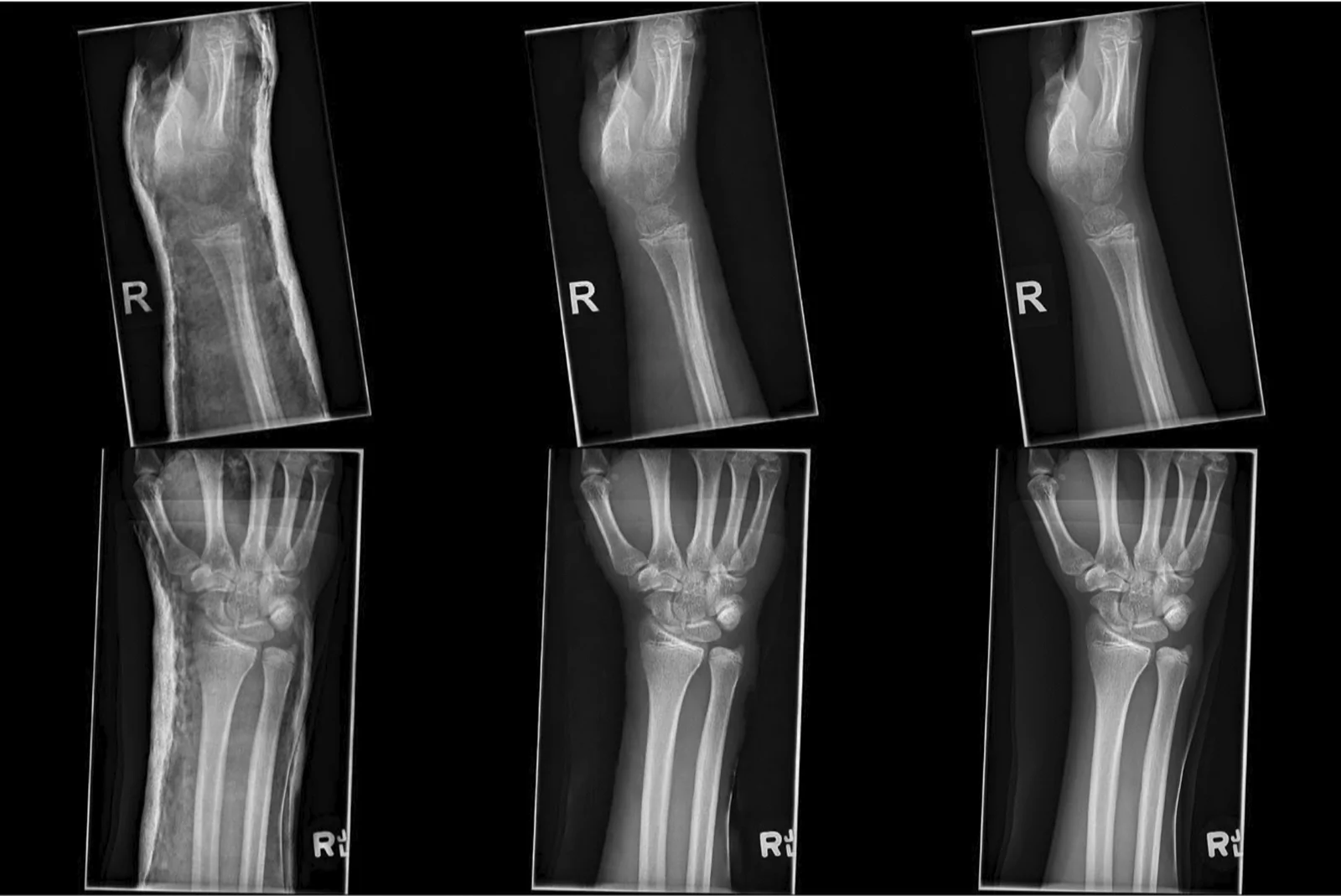
Representative examples of cast suppression applied to MELBWRI-DX test images. Top row: lateral projection. Bottom row: anteroposterior (AP) projection. Left to right: CycleGAN-generated synthetic cast image, U-Net 1024 Pix2Pix cast-suppressed image, and target cast-free reference image

Distributions of SSIM, PSNR, and MSE across the 2,000 held-out test pairs are summarised in Table 4 and visualised in Fig. 3 for each U-Net generator configuration (256, 512, and 1024). Representative examples of cast suppression using the U-Net 1024 model are shown in Fig. 4.

**Table 4:** Median (IQR) of SSIM, PSNR, and MSE values across 2,000 held-out test image pairs for each Pix2Pix U-Net generator configuration

Model	MSE (↓)	PSNR (↑)	SSIM (↑)
U-Net 256	26.8 (21.2)	33.9 (3.2)	0.952 (0.018)
U-Net 512	24.7 (17.2)	34.2 (2.9)	0.954 (0.017)
U-Net 1024	22.2 (15.8)	34.7 (2.9)	0.957 (0.017)

The U-Net 1024 configuration achieved the best overall performance, producing the highest SSIM and PSNR values and the lowest MSE. U-Net 512 achieved intermediate performance, while U-Net 256 performed the worst across all metrics. Shapiro–Wilk tests confirmed that all metric distributions deviated significantly from normality (*p* < 0.01). The Friedman test showed significant overall differences among the three model configurations for all metrics (*p* < 0.01), and post hoc Wilcoxon signed-rank tests demonstrated significant pairwise differences between every configuration after Bonferroni correction (*p* < 0.01).

### Cast suppression preprocessing experiment

The effect of cast suppression preprocessing on fracture detection performance depended strongly on the model’s exposure to cast-containing X-rays during training. For the ‘FD-0’ model, both CycleGAN and Pix2Pix preprocessing improved performance compared with the unprocessed images. Pix2Pix increased mAP@0.5 by 4.3% (0.823 to 0.858) and CycleGAN by 4.4% (0.823 to 0.859), with similar gains seen in mAP@0.5:0.95 (3.6% for Pix2Pix and 1.9% for CycleGAN). In contrast, across all models trained on cast images (‘FD-100’, ‘FD-50’, and ‘FD-25’), cast suppression consistently reduced performance, with the highest overall accuracy achieved on the original images without preprocessing. Precision–recall analysis indicated that, for models trained with cast exposure, cast suppression generally reduced precision and recall (see supplementary information).

**Fig. 5:**
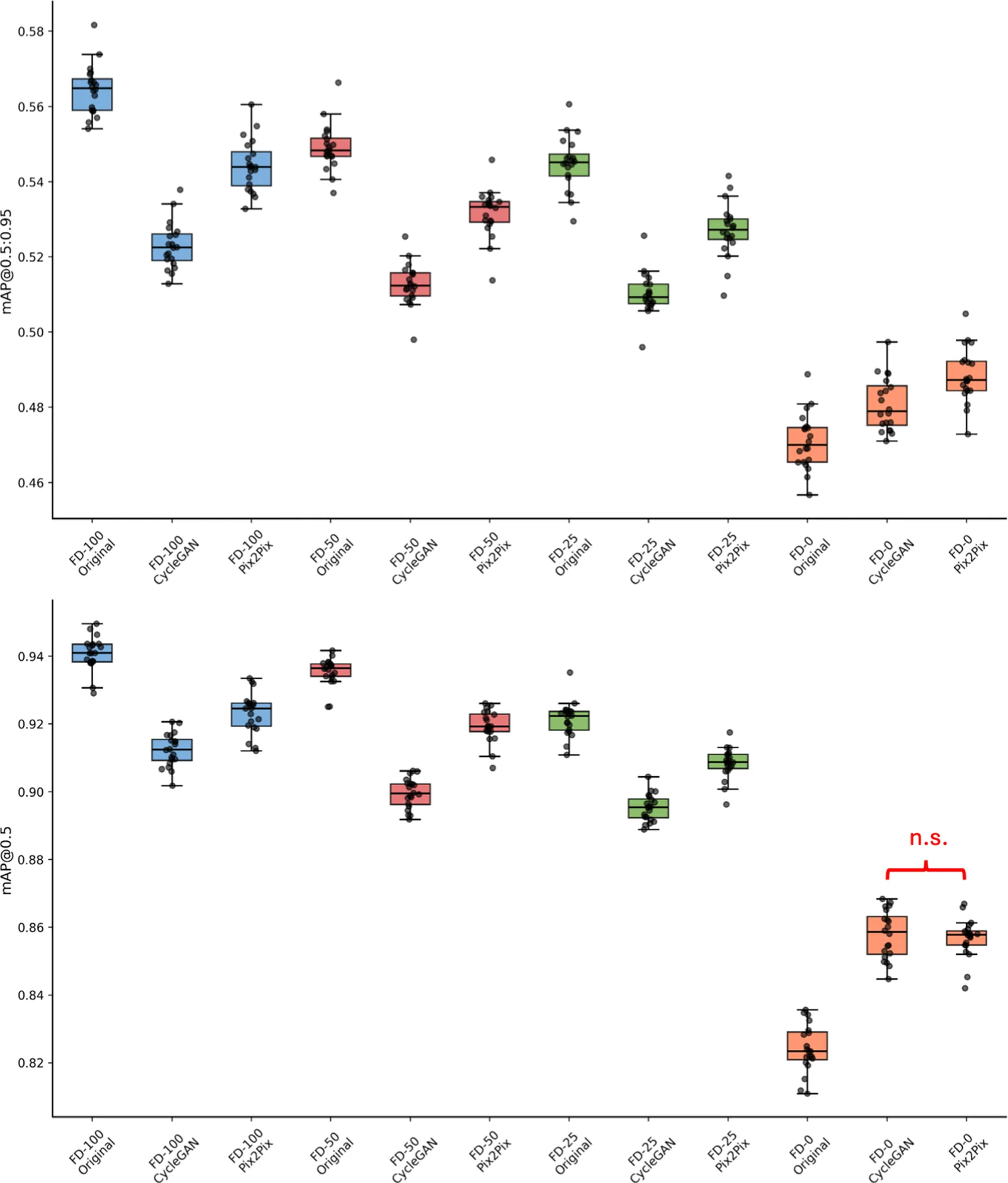
Fracture detection performance (mAP@0.5 and mAP@0.5:0.95) for the four YOLOv11-medium training configurations (‘FD-100, ‘FD-50’, ‘FD-25’, ‘FD-0’) evaluated under three preprocessing conditions (Original, CycleGAN, Pix2Pix). Box plots represent performance across 20 bootstrap test sets. Within-model pairwise comparisons showed significant differences after Holm adjustment (*p* < 0.01), except for the ‘FD-0’ model between CycleGAN and Pix2Pix (*p* = 0.39)

Across all model configurations, Pix2Pix outperformed CycleGAN, although the magnitude of improvement varied. This pattern is illustrated in Table 5 and Fig. 5. Within-model comparisons using Wilcoxon signed-rank tests with Bonferroni adjustment showed statistically significant differences between Pix2Pix and CycleGAN for most comparisons (*p* < 0.01). The only exception was within the ‘FD-0’ model, where Pix2Pix and CycleGAN did not differ significantly in mAP@0.5 (*p* = 0.39), although both significantly outperformed the unprocessed images.

**Table 5:** Median (IQR) fracture detection performance for each training configuration under Original, CycleGAN, and Pix2Pix preprocessing

Fracture detection model	Preprocessing	mAP@0.5:0.95	Change in mAP@0.5:0.95 (%)	mAP@0.5	Change in mAP@0.5 (%)
FD-100	**Original**	**0.565 (0.008)**	–	**0.941 (0.005)**	–
	CycleGAN	0.522 (0.007)	− 7.6	0.912 (0.006)	− 3.1
	Pix2Pix	0.544 (0.009)	− 3.7	0.925 (0.007)	− 1.7
FD-50	**Original**	**0.548 (0.005)**	–	**0.936 (0.004)**	–
	CycleGAN	0.512 (0.006)	− 6.6	0.899 (0.006)	− 4.0
	Pix2Pix	0.533 (0.006)	− 2.7	0.919 (0.005)	− 1.8
FD-25	**Original**	**0.545 (0.006)**	–	**0.922 (0.006)**	–
	CycleGAN	0.509 (0.005)	− 6.6	0.895 (0.006)	− 2.9
	Pix2Pix	0.527 (0.005)	− 3.3	0.909 (0.004)	− 1.4
FD-0	Original	0.470 (0.009)	–	0.823 (0.008)	–
	**CycleGAN**	0.479 (0.010)	+ 1.9	**0.859 (0.011)**	** + 4.4**
	**Pix2Pix**	**0.487 (0.008)**	** + 3.6**	**0.858 (0.004)**	** + 4.3**

Metrics shown are mAP@0.5 and mAP@0.5:0.95, calculated from 20 bootstrap evaluations. Bold values indicate the best-performing preprocessing condition for each fracture detection model and metric

**Fig. 6:**
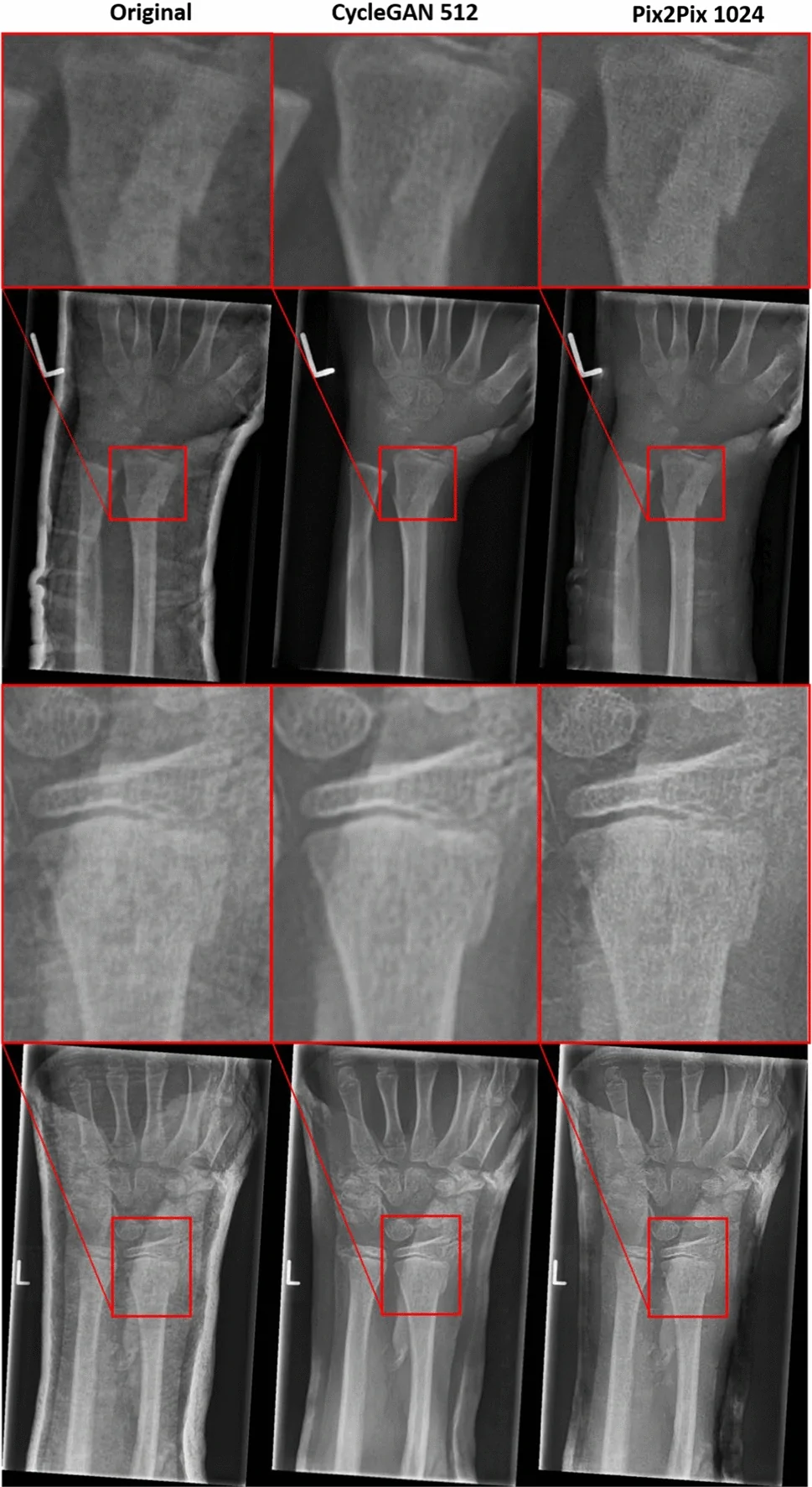
Comparative cast suppression examples across different model architectures applied to GRAZPEDWRI-DX test images. Four rows show two different wrist X-rays, each with the full image (rows 2 and 4) and corresponding fracture detail region (rows 1 and 3). From left to right: original cast image, U-Net 512 CycleGAN cast-suppressed image, and U-Net 1024 Pix2Pix cast-suppressed image, respectively. Red boxes in full images indicate the location of detailed fracture regions shown in zoomed panels

Figure 6 presents visual examples of preprocessing, illustrating CycleGAN and Pix2Pix cast suppression applied to representative GRAZPEDWRI-DX test images and highlighting the distinct texture characteristics produced by unpaired versus paired approaches. In these examples, CycleGAN substantially attenuates cast texture but is associated with noticeable smoothing and blurring of local image detail, including cortical margins and trabecular structure within the highlighted regions. In contrast, Pix2Pix tends to preserve sharper local texture and bone detail, but often leaves more residual cast material visible. These visual differences reflect the trade-off between artefact removal and preservation of fine anatomical structure.

## Discussion

Cast artefacts on follow-up wrist X-rays commonly obscure anatomical detail, complicate fracture healing assessment, and degrade automated fracture detection performance at inference when compared to cast-free images. Prior work in cast and splint suppression has relied on unpaired CycleGAN approaches [12–14], which are vulnerable to hallucination and lack robust evaluation due to the absence of true paired reference images. As a result, previous studies have been restricted to global histogram similarity metrics that do not capture spatial fidelity or labour-intensive radiologist scoring that is difficult to scale. Our study addresses these methodological limitations by generating a synthetic paired dataset that enables supervised training of Pix2Pix cast suppression models. This paired framework allows anatomically constrained image translation and supports direct pixel-wise evaluation with SSIM, PSNR, and MSE. However, neither global similarity metrics nor pixel-wise reconstruction measures can directly verify whether true anatomical structures are recovered beneath a cast, motivating evaluation using downstream tasks. We therefore evaluated how cast suppression methods influence fracture detection performance across training datasets with varying cast composition, which has not been explored in previous studies.

The performance differences between the three Pix2Pix architectures suggest that network capacity meaningfully influences cast suppression. Deeper U-Net generators have wider receptive fields and a greater ability to model both fine anatomical structures and the broader spatial patterns introduced by casts. This additional representational depth appears advantageous in a paired training setting, where the model must learn a direct mapping between synthetic cast images and original cast-free images. Although SSIM, PSNR, and MSE do not indicate clinical adequacy, they provide a more informative and meaningful assessment than the histogram-based metrics used in previous unpaired studies. These metrics therefore offer a more justified basis for selecting the most appropriate architecture for subsequent fracture detection experiments. However, high reconstruction fidelity metrics for suppression of synthetic casts do not guarantee equivalent performance on real cast artefacts, as demonstrated by the downstream fracture detection results.

Our fracture detection experiment highlights the importance of training-data composition when interpreting the effects of cast suppression as a preprocessing step. Cast suppression improved performance only for the model trained entirely without cast images, whereas models trained with cast exposure performed best on the original, unprocessed images. These results demonstrate that the impact of generative preprocessing depends critically on training data composition, suggesting that previously reported benefits of cast suppression may partly reflect the underlying bias of fracture detection models that have limited exposure to immobilisation devices during training, rather than an inherent improvement in anatomical visibility. Models trained with casts learn to accommodate their visual characteristics; removing these features at inference introduces a domain shift and may introduce artefacts that degrade performance. The previous study from Lee et al. reported improved detection performance after CycleGAN-based splint suppression [13], which contradicts our findings. Their model was trained on the FracAtlas dataset [32], which contains fractures across multiple body regions and does not label images with immobilisation devices. As a result, it is possible that their fracture detection model lacked splint-affected wrist X-rays during training, creating a distribution mismatch that could exaggerate the apparent benefit of cast suppression as a preprocessing step. Additional research is needed to determine whether cast suppression can consistently improve automated fracture detection performance across diverse datasets.

Several limitations should be considered when interpreting our findings. First, we did not perform a radiologist evaluation and therefore cannot determine whether cast suppression improves interpretation efficiency, diagnostic confidence, or visual assessment of healing in the clinical workflow. This is particularly relevant in follow-up imaging, where fractures have typically already been identified and radiologists focus on alignment, displacement, and healing rather than initial fracture detection. Our use of automated fracture detection provides an objective and scalable technical surrogate for evaluating cast suppression, but it does not directly measure clinical utility for radiologists. Nevertheless, changes in fracture detection performance provide a meaningful proxy for image quality and diagnostic information content, as substantial degradation or improvement in anatomical visibility would be expected to have concordant effects on automated detection and clinical interpretation. Second, the MELBWRI-DX institutional dataset lacked fracture annotations, preventing matched evaluation of cast suppression performance. As such, assessing the impact of cast suppression using a fracture detection model trained on and tested with an external dataset introduces a domain shift. Although this may reduce absolute performance, it tests model robustness more stringently. However, it should be noted that the combined cast suppression and fracture detection pipeline was not fully externally validated. Additionally, the synthetic paired dataset is constrained by the diversity and realism of the CycleGAN-generated casts, which may not capture the full range of cast types, materials, and placement variations encountered in clinical practice. Although combining cast-specific and splint-specific models could yield more comprehensive paired data, this would require multiple institutional datasets with immobilisation labels, which was not feasible within the scope of this study. Consequently, the achievable performance of the Pix2Pix cast suppression model is fundamentally bounded by the realism and diversity of the synthetic casts used for training.

Future research should include clinical validation with radiologist assessment to determine whether paired cast suppression improves interpretation efficiency, reduces perceptual burden, or enhances diagnostic confidence. Evidence from perceptual-education studies suggests that reducing task-irrelevant visual information can improve abnormality detection—such as the improved nodule visibility reported with limited-field-of-view chest X-rays [33]—and cast suppression may offer similar benefits by removing distracting artefacts. Additionally, image-to-image translation could serve as a data augmentation strategy to synthetically generate casts from annotated cast-free X-rays, reducing the need to annotate cast images while improving model robustness to immobilisation devices. Advanced generative approaches, such as diffusion models or physics-informed simulations using CT-derived X-rays, could produce more physically accurate cast artefacts for model development. Anthropomorphic phantom studies could generate real paired datasets for model validation. Training larger CycleGAN architectures for cast generation is not computationally feasible under limited resources, but could improve synthetic dataset quality. Finally, investigation of these methods in other anatomical regions, such as the ankle, elbow, tibia, and forearm, would be valuable.

## Conclusion

This study presents a paired image-to-image translation approach for cast suppression in wrist X-rays by generating synthetic paired data for supervised Pix2Pix training. Quantitative evaluation demonstrated that larger U-Net architectures achieve more accurate pixel-level reconstruction than smaller models. When applied as a preprocessing step to automated fracture detection, cast suppression improved performance only in models trained without cast exposure and consistently reduced performance in models trained with cast images. These findings demonstrate that cast suppression effectiveness depends critically on the downstream model’s training data composition. This work establishes a framework for developing paired cast suppression models and highlights the importance of training-data composition when integrating generative preprocessing into diagnostic pipelines.

## Supplementary Information

Below is the link to the electronic supplementary material.

Supplementary file1 (DOCX 1879 KB)
