# Supplementary material for: Enhancing automated fracture detection in paediatric wrist X-rays with paired and unpaired cast suppression methods
Source: Int J Comput Assist Radiol Surg. 2026 Mar 19;21(8):1879–90. doi: 10.1007/s11548-026-03595-2 (PMC13585798; doi:10.1007/s11548-026-03595-2)
Supplement: Supplementary file 1 [file 11548_2026_3595_MOESM1_ESM.docx]

**Supplementary Information**

**Data curation**

A YOLOv8-Large classification model was trained on the publicly available GRAZPEDWRI-DX dataset to distinguish between radiographs with and without cast artifacts. The training dataset comprised 10,000 images (5,000 cast-positive and 5,000 cast-negative) randomly selected and split into training, validation, and test sets using an 80/10/10 ratio. All images were resized to 256×256 pixels without additional data augmentation. The model was trained from scratch (without pre-trained weights) for 100 epochs using a learning rate of 0.002. On the test dataset of 1,000 images, the model achieved an accuracy of 99.5%, as demonstrated by the confusion matrix in Figure 1S.

**Fig. 1S** Confusion matrix for cast detection on the GRAZPEDWRI-DX test set (n=1,000). The YOLOv8-Large classifier achieved 99.5% accuracy, correctly identifying 496 of 500 cast images and 499 of 500 no-cast images

Following training, the model was applied to classify all 31,001 wrist radiographs, generating probabilistic predictions for cast presence. Images with p_cast_ <0.001 were selected to ensure the curation of a clean X-ray dataset for subsequent cast suppression modelling. The distribution of classification probabilities for the 31,001 X-rays is shown in Figure 2S.

**Fig.2S** Distribution of YOLOv8-assigned “no-cast” probabilities for a) the range 0 to 1, and b) for the range 0.99 to 1. The 10,000 images with the highest probabilities of no-cast were selected for subsequent cast suppression modelling

**Pix2Pix model training**

Pix2Pix models were trained using paired datasets of 10,000 aligned image pairs, each consisting of a real cast-less radiograph and a corresponding synthetic cast image generated by the CycleGAN-based pipeline. The dataset was split into 8,000 training pairs and 2,000 test pairs (80:20 split). Three generator architectures were evaluated (U-Net 256, 512, and 1024), each trained for 100 epochs (50 at a fixed learning rate of 0.002 followed by 50 with linear decay). The Adam optimiser was used with β₁ = 0.5 and a batch size of 1.

Training loss was computed as the sum of the L1 reconstruction loss and adversarial loss, weighted according to the default Pix2Pix configuration. Generator and discriminator losses were logged for all epochs to monitor convergence. Models showed stable training dynamics without mode collapse, with generator loss decreasing steadily towards the end of training and discriminator loss fluctuating within expected bounds for GAN optimisation. Training loss curves for all three Pix2Pix configurations are shown in Figure 3S.

**Fig. 3S** Training loss curves for the three Pix2Pix U-Net generator configurations (256, 512, and 1024). Generator losses are shown with L1 reconstruction and adversarial components, while discriminator loss reflects the adversarial classification task. Towards the end of training, all models exhibited stable convergence without mode collapse

**Fracture detection model training**

All four YOLOv11-medium fracture detection models were trained using identical hyperparameters. Models employed COCO-pretrained weights with conservative data augmentations, including minimal hue variance (0.01), moderate saturation (0.5) and brightness (0.3) adjustments, limited rotation (±5°), translation (0.1), scaling (0.2), and horizontal flipping (0.5). Vertical flipping, mix-up, and copy-paste augmentations were disabled to preserve anatomical fidelity. Training used the Adam optimiser with an initial learning rate of 0.001, final learning rate factor of 0.01, momentum 0.937, and weight decay 0.0005 with cosine learning rate scheduling. The loss function combined classification loss (weight 0.5), bounding box regression loss (weight 7.5), and distribution focal loss (weight 1.5). Models were trained for 100 epochs at 640×640 resolution with batch size 16, employing a 3-epoch warmup and disabling mosaic augmentation in the final 10 epochs. Training curves demonstrated consistent convergence across all dataset compositions, with final validation losses stabilising by epoch 80. Loss curves for all fracture detection models are presented in Figure 4S.

**Fig. 4S** Training and validation metrics for the six YOLOv11-medium fracture detection models: a) FD-100, b) FD-25, c) FD-50, and d) FD-0. Each subplot displays the evolution of bounding box regression loss, classification loss, and distribution focal loss for both training and validation sets, alongside overall dataset precision, recall, mAP@0.5, and mAP@0.5:0.95 (across all 9 classes in GRAZPEDWRI-DX). All models exhibited stable optimisation behaviour, with losses converging and detection metrics plateauing by approximately epoch 80, indicating consistent convergence across dataset compositions

**Statistical analysis**

For the cast suppression preprocessing experiment, the performance variability of each fracture detection model was estimated using a non-parametric bootstrap. Twenty bootstrap test sets were generated by sampling with replacement from the original 3,043-image test set, each preserving the same sample size (N = 3,043). As shown in Figure 5S, the number of bootstrap resamples was selected based on an empirical convergence analysis, demonstrating that fracture detection performance metrics stabilise beyond 20 bootstrap iterations.

**Fig. 5S** Empirical bootstrap convergence analysis for fracture detection performance. Running medians and interquartile ranges of mAP@0.5 and mAP@0.5:0.95 are shown as a function of the number of bootstrap resamples (k), for each fracture detection training configuration (FD-100, FD-50, FD-25, FD-0). Performance estimates exhibit minimal change beyond approximately 20 bootstrap resamples, supporting the choice of N = 20 for the main analysis

**Cast suppression preprocessing experiment**

In addition to mAP-based evaluation, precision–recall behaviour was examined to assess whether cast suppression disproportionately affected false-positive or false-negative detections. Precision–recall analysis (Figure 6S) showed that, for models trained with cast exposure (FD-100, FD-50, FD-25), cast suppression generally reduced precision and recall, indicating increases in both false positives and false negatives rather than a selective shift in one error type. In contrast, for the FD-0 model, cast suppression was associated with improved recall, consistent with the mAP findings.

**Fig. 6S** Precision and recall distributions for fracture detection models trained with varying cast exposure (FD-100, FD-50, FD-25, FD-0) evaluated under Original, CycleGAN, and Pix2Pix preprocessing. Box plots summarise precision and recall across 20 bootstrap test sets
